# Social prescribing for people recovering from treatment for cancer: a systematic scoping review

**DOI:** 10.1007/s00520-026-10561-w

**Published:** 2026-04-25

**Authors:** Jazlan Jamaluddin, Katerina Nikitara, Shrikant Atreya, Sajaratulnisah Othman, Ferdinando Petrazzuoli, Roiyah Saltus, Joyce Kenkre

**Affiliations:** 1 Department of Primary Care Medicine, Faculty of Medicine, Universiti Malaya, Kuala Lumpur, Malaysia; 2 Hellenic Cancer Federation (ELLOK), Solonos St. 94, 10680 Athens, Greece; 3 Department of Palliative Care and Psychooncology, Tata Medical Centre, Kolkata, India; 4 WONCA Special Interest Group for Palliative Care and Cancer, Brussels, Belgium; 5 WONCA Special Interest Group for Family Violence, Brussels, Belgium; 6 Department of Clinical Sciences in Malmö, Centre for Primary Health Care Research, Lund University, Malmö, Sweden; 7 European Rural and Isolated Practitioners Association (EURIPA), Paris, France; 8 Faculty of Life Sciences and Education, University of South Wales, Pontypridd, UK; 9 WONCA Special Interest Group for Social Prescribing and Community Orientation, Brussels, Belgium

**Keywords:** Social prescribing, Cancer survivors, Complementary therapies, Psychological well-being, Quality of life

## Abstract

**Background:**

Social prescribing (SP) has emerged as a non-medical strategy to enhance cancer survivors’ well-being by addressing psychosocial challenges through diverse interventions. Despite growing evidence of benefits, a comprehensive synthesis of intervention effectiveness and implementation barriers remains limited. This scoping review updates current evidence on SP interventions, outcomes, and challenges among cancer survivors.

**Purpose:**

To identify and map SP interventions for post-treatment cancer survivors, assess their impact on well-being and quality of life, and summarise barriers and recommendations for improved accessibility and sustainability.

**Methods:**

Peer-reviewed studies involving adult cancer survivors that evaluated SP-related interventions with psychological, emotional, or quality of life outcomes were included. Reviews, editorials, conference abstracts, and non-English studies were excluded. Three databases (Scopus, PubMed, APA PsycINFO) were searched up to March 31, 2025. Data extraction captured study design, intervention type, setting, population, outcomes, and reported benefits or limitations. Narrative synthesis followed PRISMA-ScR and SWiM guidelines.

**Results:**

Thirty-two studies assessed seven intervention types: gardening, peer support, expressive writing, art therapy, physical activity, blue prescriptions, and spiritual care. These interventions enhanced emotional resilience, reduced stress, fostered social connections, and improved coping. Reported barriers included limited access to resources, participant engagement, professional support, and long-term sustainability.

**Conclusion:**

SP interventions provide significant psychological and emotional benefits for cancer survivors. Addressing accessibility, engagement, and sustainability challenges is essential. Future research should explore hybrid and digital SP models with structured professional involvement to improve reach and effectiveness.

**Supplementary Information:**

The online version contains supplementary material available at 10.1007/s00520-026-10561-w.

## Introduction

Cancer is the second leading cause of death globally [1–3]. In response, the European Union (EU) Mission on Cancer aims to improve the quality of life for three million people by 2030 through prevention, treatment, and rehabilitation, enabling them to live longer and healthier lives [4]. Policymakers at both the EU and state levels seek to enhance civic engagement across diverse societal groups while addressing cancer and its associated consequences, including the effects of cancer treatments. Social prescribing (SP) has been increasingly positioned within European and UK policy discourse as a mechanism to connect individuals with non-clinical community resources to support recovery and long-term well-being [5, 6]. SP involves referring individuals with social, emotional, or practical needs to non-clinical support services within community settings to improve mental and physical well-being. In cancer survivorship, SP initiatives may target multiple domains including psychological well-being (e.g. stress reduction, coping, and emotional resilience), physical functioning (e.g. movement-based or rehabilitation-supportive activities), and social participation (e.g. reducing isolation and enhancing community connection) [7, 8]. Activities are often classified based on thematic colour codes (e.g. green for nature-based interventions, such as urban gardening and walking, and blue for water-related activities, including swimming or wetland conservation), the nature of participation (individual vs. group-based), or cultural underpinnings, including heritage-focused programmes like museum visits and choir participation. Additionally, SP initiatives may be classified according to their setting, either within clinical environments involving healthcare providers and link workers or in community-based settings facilitated by local organisations. Recent research has distinguished between the conceptual definition of SP (a person-centred approach to address non-medical determinants of health), its intended meaning (a mechanism for connecting individuals to community resources through referral or recommendation), and its operational components (identifiable processes such as assessment of needs, structured referral pathways, link-worker or facilitator involvement, and measurable outcomes such as well-being, quality of life, and social connectedness) [9]. In practice, SP commonly involves an intermediary step in which individuals’ needs are assessed, and they are connected to appropriate community-based resources, although terminology and operational reporting remain inconsistent across health systems and research settings, and similar interventions may be described using alternative labels [10].

The underlying premise of SP is that not all health needs necessitate pharmacological or medical interventions. Instead, many individuals benefit when their social, emotional, and practical needs are addressed, leading to improved health and well-being. This approach leverages community and cultural resources that serve as protective factors against the broader determinants of health, offering individuals greater control over their well-being. There is growing evidence that SP can include nature-based interventions, often delivered through third-sector or community organisations, and that these can contribute to physical, psychological, and social well-being. For example, “green SP” programmes connect people to community gardening, walking schemes, conservation volunteering, blue-space activities, and other outdoor options to improve mental and physical health and reduce loneliness, with national programmes in England reporting improvements in well-being and engagement among diverse populations [11]. A recent systematic review and meta-analysis also support the inclusion of nature-based social prescriptions in mental health care plans and highlight their potential to improve psychological outcomes [12, 13]. However, despite increasing interest and policy support, evidence on specific operational models and long-term health outcomes remains limited, particularly in oncology contexts [14].

Within this broader context, and as part of a larger research programme exploring the role, value, and impact of volunteering across Europe, this scoping review will examine the effect of SP on cancer patients recovering following treatment. The overarching aim of this scoping review is to map the existing evidence on community-anchored social prescribing interventions for post-treatment cancer survivors and to summarise their reported impacts on health and well-being. In addition, we sought to identify commonly reported implementation challenges, barriers, and evidence gaps relevant to the development and sustainability of SP within cancer survivorship pathways. By synthesising the available literature, this review provides an overview of the range of SP-relevant interventions currently described, the outcomes assessed, and areas where further research is needed to strengthen evidence for SP in oncology survivorship care.

## Methods

### Protocol

This scoping review was conducted based on a systematic methodology in adherence to the PRISMA (Preferred Reporting Items for Systematic Reviews and Meta-Analyses) and Synthesis Without Meta-analysis (SWiM) framework [15, 16]. A protocol for this scoping review was prospectively registered on the Open Science Framework (OSF) on 22 September 2025 (10.17605/OSF.IO/TDNSH). The eligibility of studies was determined using the PEO framework (Population, Exposure, Outcome). The population included adult cancer survivors (≥ 18 years) who had completed primary cancer treatment, regardless of cancer type. Primary treatment was defined as surgery, chemotherapy, radiotherapy, immunotherapy, targeted therapy, hormone therapy, or transplantation as part of cancer management, and survivors could be at any stage of recovery following completion of these treatments. The exposure comprised community-anchored social prescribing interventions, defined as structured referral or recommendation pathways linking survivors to non-clinical or community-based resources (e.g. initiated by a healthcare professional, programme facilitator, or organised survivorship service). Interventions could be delivered either face-to-face or remotely and were required to address non-medical supportive needs across psychological, social, or physical well-being domains. The outcomes assessed included quality of life, physical and psychological health and well-being, social inclusion, and disease-specific measures. Both qualitative and quantitative study designs were included, whereas reviews, conference abstracts, opinion papers, correspondence, perspectives, letters, and editorials were excluded. No restrictions were applied regarding the timeframe, and only studies published in English were considered. Grey literature and non-English records were excluded due to resource constraints. Although grey literature is highly relevant to social prescribing given its policy- and practice-oriented nature, a comprehensive grey literature search was not feasible within the available time and resources. Grey literature sources are diverse and often inconsistently indexed and would have required additional systematic searching, screening, and verification processes beyond the scope of this review. Therefore, this review was restricted to peer-reviewed publications to ensure the feasibility and consistency of evidence identification. This exclusion is acknowledged as a limitation, and future reviews should incorporate grey literature to provide a more complete map of social prescribing policy and implementation evidence.

To ensure conceptual alignment with SP, included interventions were required to be community-based or community-anchored (e.g. delivered through community organisations, voluntary sector services, peer-led groups, or community facilities) and linked through a structured referral or recommendation pathway to a non-clinical resource (e.g. initiated by a healthcare professional, programme facilitator, or organised survivorship service) [17, 18]. Studies were not required to explicitly use the term “social prescribing”; however, standalone psychosocial or exercise interventions delivered solely within clinical survivorship research without a referral or prescribing component were excluded, which distinguishes this review from the broader psychosocial and exercise oncology literature.

### Search strategy

Peer-reviewed publications were identified through a comprehensive literature search across three electronic databases: Scopus, PubMed (MEDLINE), and APA PsycINFO. The search covered literature published up to March 31, 2025, and no further updates were performed after that date. Furthermore, the reference lists of studies included at the full-text review stage, as well as previous reviews identified during the search, were examined for additional relevant studies. The search strategy and terms used were shown in Supplementary 1. The search strategy was developed iteratively based on the review objectives and key concepts of cancer survivorship and SP, including commonly used substitute terms (e.g. community referral, link worker, community connector, non-clinical interventions, voluntary sector support, social support, and community-based interventions). An initial limited exploratory search was undertaken to identify relevant keywords and index terms, which informed refinement of the final search strings. Searches were conducted from database inception to March 2025, and the strategy was adapted for each database according to its indexing system and syntax. The final searches were conducted on March 31, 2025, and no further updates were performed after March 2025. We acknowledge that SP terminology and pathway components (e.g. link workers, referral mechanisms, and structured community linkage models) are inconsistently reported in the titles and abstracts of survivorship interventions across countries and disciplines; therefore, broader terms were included to maximise sensitivity and reduce the risk of missing interventions that may be operationally consistent with SP but not explicitly labelled as such. Conceptual alignment with SP was subsequently ensured during screening by applying eligibility criteria requiring a community-anchored intervention linked through a structured referral or recommendation pathway to a non-clinical resource. The database selection was guided by feasibility and the aim to capture multidisciplinary evidence across biomedical, psychological, and public health literature; however, we acknowledge that additional databases such as Embase, CINAHL, CENTRAL, and Web of Science may contain further relevant studies and should be considered in future updates of this review.

### Study selection

Studies retrieved from electronic searches were entered into Rayyan (a web-based tool for systematic review screening) for de-duplication and screening based on the review’s inclusion criteria [19]. Initially, all studies were assessed based on titles and abstracts. A pilot title and abstract screening was independently conducted by three reviewers on 10% of the identified studies. Discrepancies were resolved through discussion or with the involvement of an external reviewer. Agreement between reviewers was estimated using percentage agreement. If agreement was below 90%, the inclusion criteria were reviewed and refined, followed by an additional round of independent double-screening to reassess concordance. Upon reaching a 90% agreement threshold, the remaining articles were screened separately by two reviewers. Due to the high volume of records excluded during the title and abstract screening stage (*n* = 9335), detailed reasons for exclusion at this stage were not systematically recorded. This decision was made to ensure the feasibility of the screening process within the available time and resources. Reasons for exclusion were documented in detail only during full-text eligibility assessment. Full texts of studies meeting the inclusion criteria were subsequently reviewed independently by four reviewers. During full-text screening, reviewers assessed eligibility against the predefined PEO criteria, including confirmation that participants were post-treatment cancer survivors, that the intervention was community-based or community-anchored, and that it was linked through a structured referral or recommendation mechanism consistent with SP. Reasons for exclusion at the full-text stage were documented and categorised (e.g. wrong study design, wrong population, wrong intervention). Any disagreements were resolved through discussion among the review team until consensus was achieved.

### Data extraction and presentation

Data extraction was guided by the Joanna Briggs Institute (JBI) methodological framework for scoping reviews. Data extraction was performed using a standardised and piloted extraction form in Microsoft Excel, capturing details on study design, setting, sample characteristics, types of interventions, and assessed outcomes. All authors independently extracted and reviewed the data, with any discrepancies resolved through collective discussion. The results were then presented in a structured format. Extracted data including author, year, and country of publication are summarised in Table 1. These details were not repeated consistently throughout the narrative synthesis in order to reduce redundancy and maintain readability. We grouped studies by intervention types (gardening, peer support, art therapy, expressive writing, yoga/spiritual, nature-based, physical activity) and narratively synthesised effects across common outcome domains (quality of life, psychological distress, fatigue, function, social connectedness). Effect direction was judged relative to each study’s prespecified primary outcome(s); where outcomes conflicted, we prioritised validated multi-domain instruments (e.g. FACT-G, SF-36). Consistent with PRISMA-ScR and JBI guidance, we did not conduct a formal risk-of-bias appraisal because the objective was to map the breadth and nature of evidence rather than estimate effects.

**Table 1:** Summary of the characteristics of the included studies involving social prescribing among people recovering from cancer treatment

First author, year	Location	Study design	Population	Sample size	Intervention (duration, setting, provider)	Outcomes measured	Key outcomes
Gardening interventions							
Wendy Demark-Wahnefried, 2024 [20]	Alabama, USA	RCT	Medicare-eligible cancer survivors	381 (194 intervention)	1-year mentored home gardening (home-based; Master Gardeners, Alabama Extension)	Two-year physical function, diet, and microbiota	No significant improvement in composite index of diet, activity, and physical function
Harsh Sharma, 2022 [21]	Alabama, USA	Feasibility study	Cancer survivors	30	9-month home gardening (home-based; Cooperative Extension Service)	QoL and Physical Functioning (PF)	Reduced depression, fatigue; improved physical function
Jennifer R. Bail, 2018 [22]	Birmingham, USA	RCT	Breast cancer survivors	82	1-year mentored gardening (home-based; Master Gardeners)	Physical function, vegetable intake, biomarkers	RCT was modified and delayed due to COVID-19
Wendy Demark-Wahnefried, 2018 [23]	Alabama, USA	RCT	Elderly cancer survivors	46	1-year mentored gardening (home-based; Cooperative Extension Master Gardeners)	Feasibility (accrual, retention, safety). Diet, physical activity/performance, anthropometrics, and psychosocial health	Increased reassurance of worth, vegetable intake; reduced waist circumference growth
Cindy K. Blair, 2013 [24]	Alabama, USA	Feasibility study (single-arm)	Adult/child cancer survivors	12	Mentored gardening (home-based; Master Gardeners)	Feasibility, recruitment, satisfaction	Improved physical function; 40% increased vegetable intake
Renate M. Winkels, 2019 [25]	Pennsylvania, USA	Feasibility study (mixed-methods)	Adolescent and young adult (AYA) cancer survivors	5 (4 completed)	Community gardening (hospital garden; Master Gardeners)	Diet, physical activity, psychosocial	Feasible for AYA survivors
Cindy K. Blair, 2021 [26]	New Mexico, USA	Feasibility study	Cancer survivors in New Mexico	30	9-month mentored home gardening (home-based; Cooperative Extension Master Gardeners)	Accrual, retention, satisfaction, fruit/vegetable intake, physical activity, and quality of life	The programme is adapted in New Mexico
Mallory G. Cases, 2022 [27]	Alabama, USA	Mixed methods	Cancer survivors	46 (30 in qualitative)	1-year mentored home gardening (home-based; Cooperative Extension Master Gardeners)	Continued gardening, garden expansion, and organisational sustainability capacity (PSAT scores)	Most continued gardening; programme rated highly but funding lowest
Peer support							
Nicholas et al., 2012 [28]	Toronto, Canada	Mixed-methods (questionnaires and qualitative interviews)	Fathers of brain tumour children	21	Online peer support (online; moderated group)	Paternal coping, isolation, and quality of life	Improved paternal coping. Benefited from networking, emotional expression
Stegina et al., 2005 [29]	Australia	Quantitative (cross-sectional survey)	Prostate cancer patients	1,224	Face-to-face peer support meetings (support group setting; peer-led)	Satisfaction (PCSI), QoL, distress, and pain	High satisfaction is related to better QOL and lower pain
Huber et al., 2018 [30]	Germany	Quantitative (cross-sectional comparison study)	Prostate cancer patients	1,117	Online vs face-to-face peer support (online + in-person; peer-led)	Decision-making, distress, and QoL	Online groups reported higher distress. No differences in anxiety, depression, and global QoL
Morris et al., 2012 [31]	Australia/USA	Quantitative (prospective survey)	Breast cancer survivors	51	1,000-mile group motorcycle ride (group challenge; peer-led)	Posttraumatic growth (PTG) and distress	Reduced distress; increased post-traumatic growth
Yoga/spiritual							
David Victorson, 2024 [32]	USA	Single-arm pilot study (mixed-methods)	Latine cancer survivors	35	12-week yoga (community-based; bilingual instructors/researchers)	Feasibility, QoL, physical function, and stress	Improved sleep, pain interference, depression, blood pressure
Ashley N. Ross Zahavich, 2013 [33]	Canada	14-week feasibility study (pre-post design)	Prostate cancer survivors	15	7-week yoga (university fitness facility; yoga instructors/researchers)	Feasibility, physical activity levels, QoL, and mood	Reduced stress, fatigue, and mood
Mary Lou Galantino, 2012 [34]	USA	Exploratory qualitative study	Breast cancer survivors	10	8-week yoga (clinical/community; PT + yoga instructors)	Joint pain (arthralgia) and QoL	Improved QoL and reduced arthralgia pain, improved physical fitness
Marieke Van Puymbroeck, 2013 [35]	USA	Qualitative (interpretative phenomenological analysis)	Breast cancer survivors	18	8-week yoga (hospital-based; yoga therapist)	Perceived health benefits (focus groups)	Improved physical/mental health and social health/healing
Suzanne C. Lechner, 2014 [36]	USA	Randomised controlled trial (RCT)	Black breast cancer survivors	114	10-week stress management/CBSM (community-based; trained facilitators)	Psychological adaptation (stress, depression, growth)	Both improved QoL. No significant difference between two groups
Creative							
Comello MLG, 2016 [37]	Online	Cross-sectional	Cancer survivors	794	Recreational gaming (online; self-administered)	Self-efficacy, resilient coping, and flourishing	Improved self-efficacy, coping
Reynolds F, 2007 [38]	England	Qualitative (Interpretative Phenomenological Analysis)	Women with cancer	12	Visual art-making (community/leisure; self-directed)	Subjective well-being and identity meanings	Artmaking supported well-being through positivity, identity, social connection, and emotional expression
Chan NCT, 2022 [39]	Hong Kong	Mixed-methods	Breast/gynaecological cancer	54	8-week art therapy (community cancer service; art therapist)	Emotional distress (distress thermometer) and mental wellness (C-SWEMWBS)	Six loss/readjustment themes found. Reduced distress, improved mental well-being
Warson E, 2012 [40]	USA	Mixed-methods pilot study	Native cancer survivors	46	Culturally relevant art therapy (tribal/urban tribal centre; provider unclear)	Stress reduction (STPI) and artwork analysis	Quantitative results inconclusive; Qualitative coding showed holistic wellness concept
Öster RNT, 2008 [41]	Sweden	Qualitative (based on a previous RCT)	Breast cancer survivors	42	Art therapy (radiotherapy dept; nurse/art therapist)	Meaning-making regarding gendered boundaries; verbal reflections	Themes identified: Being someone who reacts, connects body/self, locates, sees, acknowledges
Expressive writing							
Qiao Chu, 2019 [42]	USA	RCT	Chinese American breast cancer survivors	96	3-week expressive writing (self-administered; researcher-guided)	PTSD symptoms (re-experiencing, avoidance, arousal)	Reduced PTSD symptoms
Qian Lu, 2018 [43]	USA	RCT	Chinese-speaking breast cancer survivors	136	Culturally tailored writing (self-administered; researcher-guided)	Quality of Life (FACT-G) at baseline, 1, 3, and 6-month follow-ups	Improved QoL
Krystal Warmoth, 2019 [44]	USA	Pilot study (using CBPR approach)	Chinese American breast cancer survivors	39	Psychosocial program (community-based; culturally matched mentors/educators)	Post-traumatic growth, positive affect, and appreciation for life	Improved positive affect and growth
Qiao Chu, 2020 [45]	USA	RCT	Chinese American breast cancer survivors	136	Expressive writing (self-administered; researcher-guided)	PTSD symptoms at 1, 3, and 6-month follow-ups	Reduced PTSD symptoms. Social constraints affect outcomes
Qian Lu, 2012 [46]	USA	Pilot study (using CBPR approach)	Chinese-speaking breast cancer survivors	19	Expressive writing (community-based; researchers/community partners)	QoL, fatigue, PTSD symptoms, intrusive thoughts, and positive affect	Improved QoL, fatigue
Nature-based							
Arnau Carreno, 2023 [47]	Spain	Mixed-methods pilot study	Cancer survivors	16	Sea bathing/swimming (coastal blue spaces; medical + sea experts)	Heart rate, sleep quality, and mood states (POMS)	Improved mental health. Reduced tension and anger; improved vigour
Lisa Kuballa, 2023 [48]	Germany	Non-randomised controlled study	Cancer patients	107	12-week nature programme (hospital park; integrative medicine team)	Quality of Life (FACT-G) and fatigue levels	Improved QoL, fatigue and psychological parameters
Maiko Nakau, 2013 [49]	Japan	Pilot study (quantitative focus)	Breast/lung cancer	22	12-session nature therapy + yoga/meditation (urban green space; integrative medicine team)	Spirituality (FACIT-Sp) and Quality of Life (SF-36)	Improved QoL, reduced fatigue, improved psychological state. Increased natural killer cell activity
Physical activity							
Knobf, 2014 [50]	New Haven, USA	1-group pretest–posttest design	Breast cancer survivors	26	4–6-month gym programme (community fitness centres; supervised trainers)	Physical/psychological symptoms and Quality of Life	Improved QOL and functional ability, reduce fatigue/depression (75–98% adherence)
Fischer, 2015 [51]	Netherlands	Pre-post intervention study	Breast cancer survivors	28	10-week Nordic walking (outdoor paths; PT + oncology specialists)	Illness perceptions (BIPQ) and social functioning	Increase vitality/shoulder mobility; Decreased perceived shoulder symptom severity and daily limitations. 9/10 satisfaction

## Results

### Overview of the studies

**Fig. 1:**
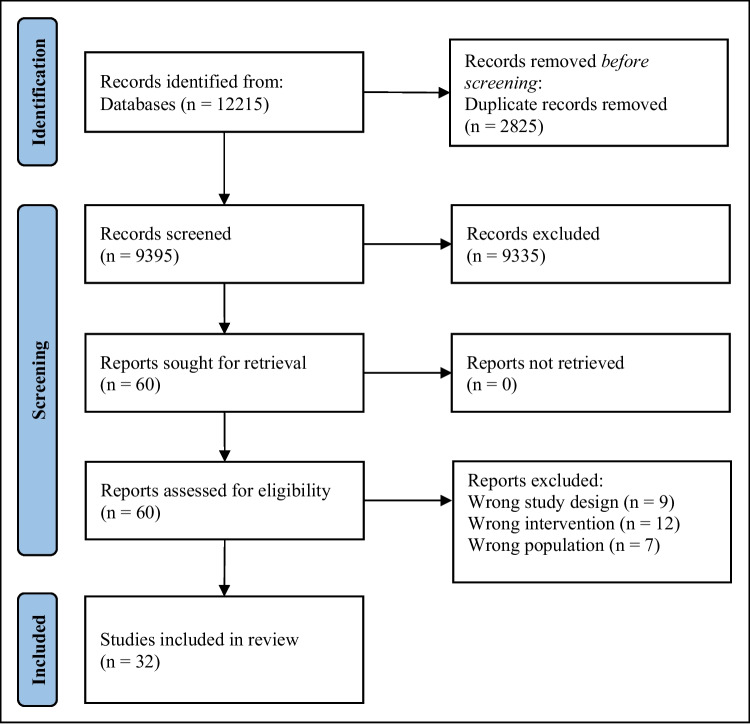
Preferred reporting items for systematic reviews and meta-analyses (PRISMA) flow diagram of this review

The electronic searches yielded 12,215 citations. A total of 60 published studies were reviewed after deduplication and initial screening, of which 32 met the inclusion criteria (Fig. 1). Studies excluded at full-text review were categorised as follows: wrong study design (*n* = 9), which included reviews, editorials, protocols, conference abstracts, and non-primary research; wrong intervention (*n* = 12), which included interventions not aligned with a community-based or non-clinical social prescribing approach or lacking relevant well-being outcomes; and wrong population (*n* = 7), which included studies not involving post-treatment cancer survivors (e.g. participants undergoing active treatment or non-cancer populations). Table 1 summarises the included studies and reports study methodology (quantitative, qualitative, or mixed-methods), sample size, intervention setting, intervention provider/professional group (where reported), and key findings. There were seven broad intervention types identified: gardening (*n* = 7), peer support (*n* = 5), creative interventions (*n* = 5), yoga/spiritual prescription (*n* = 5), expressive writing (*n* = 5), nature interventions (*n* = 3), and physical activity interventions (*n* = 2). These interventions were delivered in a range of community and non-clinical settings and assessed outcomes including quality of life, psychological well-being, social connectedness, and physical functioning. In the following section, a brief overview of the interventions and their outcomes is given.

### Gardening interventions

Between 2010 and 2022, multiple studies examined *Harvest for Health*, a home-based vegetable gardening programme pairing cancer survivors with Cooperative Extension–certified Master Gardeners across several US regions [20, 21, 23, 26, 27]. The intervention was informed by Social Cognitive Theory and the Social Ecological Model and aimed to improve diet, physical activity, and well-being. An initial Alabama feasibility study reported improvements in functional performance (e.g. grip strength and chair-stand repetitions), increased fruit and vegetable intake by at least one serving per day, and increased weekly physical activity of at least 30 min [24]. A randomised feasibility trial among breast cancer survivors replicated these findings, showing significant improvements in moderate physical activity and strength tests, high satisfaction, and 86% retention at 2-year follow-up; the programme was especially appealing to participants with travel limitations [22]. A larger feasibility trial among older survivors of mixed cancer types incorporated accelerometry, standard physical performance batteries, and biomarkers including interleukin-6, telomerase activity, and cortisol; participants increased fruit and vegetable consumption by one serving per day (*P* = 0.02) and reported improved self-worth (*P* = 0.02), with mixed effects on waist circumference and quality of life, warranting further exploration [23]. Sustainability analyses revealed robust long-term engagement: 85.7% of participants continued gardening beyond the intervention, and nearly half expanded their plots, while stakeholders rated sustainability highly on the Program Sustainability Assessment Tool (mean 5.7/7), although funding stability remained uncertain [27]. A large crossover randomised controlled trial with 381 Medicare-eligible survivors found no change in a composite index combining diet, physical activity, and physical function, yet noted meaningful improvements in individual domains such as fruit and vegetable intake, physical performance, perceived health, and gut microbiome diversity [23]. Pandemic-adapted implementations maintained feasibility: participants increased fruit and vegetable intake (for example, + 1.2 servings/day), reported stable or improved social functioning, and expressed high satisfaction (93–100%), frequently citing mindfulness and connection to nature as salient benefits [21, 52]. A small mentored gardening programme for adolescent and young adult survivors demonstrated successful planting and harvesting, greater vegetable intake, and enjoyment, but also highlighted logistical barriers such as scheduling conflicts and weather, and recommended more structured education, longer seasons, and enhanced peer interaction [25]. Across gardening studies, samples skewed toward women and breast cancer survivors, with older adults overrepresented; design heterogeneity, absent cost-effectiveness analyses, pandemic-related disruptions, and a lack of long-term clinical endpoints such as recurrence or survival qualify interpretation. Nonetheless, the individualised, home-based model consistently achieved high engagement and retention and appears feasible and acceptable for enhancing diet, physical activity, and psychosocial outcomes, meriting research on cost, broader rollout, and mechanisms, particularly in underserved and aging populations.

### Peer support interventions

Four studies evaluated peer support interventions directed at survivors and caregivers, focusing on prostate and breast cancer populations and caregivers of paediatric brain tumour patients, with adult beneficiaries typically aged 49 to 68 years. Definitions of survivorship varied: fathers of paediatric brain tumour survivors within 6 months to 5 years post-treatment were included in one study [28], prostate cancer survivors 12 months to more than 3 years post-diagnosis in another [29], while two studies simply required completion of cancer-specific treatment [30, 31]. Formats ranged from group discussions with educational components and follow-up via phone and newsletters [29], to small groups led by trained peer facilitators from prostate cancer organisations covering emergency recognition, first aid, treatment planning, and strategies to access medical and emotional support [30], to an experiential 10-day motorcycle adventure for breast cancer survivors riding 100–180 miles per day, with motorcycles donated and participants fundraising for fuel and lodging [31]. Across these diverse models, participants described reduced distress, strengthened self-reliance, restored sense of control, deeper emotional bonding, diminished isolation, and a safe space to express fear, guilt, and anxiety [28, 31]. Peer engagement also improved decision-making autonomy and communication with clinicians, particularly among younger, educated survivors from higher socioeconomic strata with fewer physical symptoms and stronger physician support [29, 30]. Key limitations include participants’ desire for more direct healthcare professional input on medical issues [28], limited attention to caregivers despite their central role (only one study emphasised caregiver experience) [28], probable selection bias toward individuals with better baseline coping, and the need to optimise low-burden, cost-effective, and sustainable delivery models. Even with these caveats, peer support consistently enhanced psychological well-being and social connectedness, though evidence remains limited by heterogeneity and non-randomised designs.

### Yoga/spiritual interventions

Five studies examined yoga and stress-management interventions, predominantly among breast and prostate cancer survivors and spanning 2012–2024. Four focused on yoga [32–35], and one on community-based cognitive-behavioural stress management (CBSM) [36], with a clear trend toward culturally sensitive, community-anchored delivery. A 12-week Spanish-language programme tailored for Latina survivors used culturally appropriate materials and built structured social time into each session [32]. Two 8-week programmes blended in-class and at-home practice emphasising empowerment and healing, relying on qualitative methods such as focus groups, journals, and observations to capture lived experience [34, 35]. To improve adherence, another intervention paired a 7-week self-guided physical activity phase with a subsequent 7-week group yoga phase and evaluated biometric and psychosocial outcomes [33]. Across methodologies, participants reported gains in physical function, posture, strength, flexibility, mood, stress reduction, relaxation, and social connectedness, with communal participation consistently reinforcing engagement. In underserved Black breast cancer survivors, community-based cognitive-behavioural stress management was associated with improved psychological adaptation and quality of life outcomes, although differences between intervention formats were not statistically significant.

### Creative interventions

Five creative-arts studies from 2001 to 2018, using qualitative, quantitative, and mixed methods across varied cultural and clinical contexts, demonstrate that art therapy supports psychological well-being, emotional resilience, and identity reconstruction. Personal art-making enabled women with cancer to disengage from illness narratives and reclaim social and personal identity [38]; expressive art sessions allowed survivors to challenge normative femininity and reassert agency through self-representation [41]; and an 8-week culturally adapted programme for women recovering from breast and gynaecologic cancers reduced distress and improved well-being in an emotionally safe environment [39]. Creative expression also extended beyond traditional arts: a cross-sectional survey of 794 survivors linked intrinsic gaming motivations such as curiosity and mastery with communication self-efficacy, resilient coping, and flourishing, illustrating that therapeutic creativity can include recreational digital gaming [37]. In Indigenous contexts, workshops grounded in holistic wellness fostered spiritual and communal healing but exposed the inadequacy of standardised psychological measures not aligned with cultural values [40]. Across studies, participants described empowerment, catharsis, and affirmation of identity, with common mechanisms including safe expression, reconnection with self, and supportive spaces. Limitations include small samples, non-randomised designs, lack of control groups, and measurement challenges for subjective, culturally embedded outcomes. Future research should prioritise culturally attuned designs, larger cohorts, and rigorous, context-appropriate evaluation to clarify the impact on survivorship trajectories.

### Expressive writing interventions

Five studies focused largely on Chinese American breast cancer survivors in their fifties, spanning pilot work in 2012 to randomised designs by 2020 [31–35]. A 3-week protocol emphasising emotional disclosure, coping, and benefit-finding improved quality of life and post-traumatic growth while reducing post-traumatic stress symptoms [35]. Moderation by acculturation and social constraints was observed, with higher social constraint predicting greater symptom reduction following expressive writing [42–45]. A mixed-methods programme that combined peer mentoring, education, and native-language support reinforced social engagement and emotional resilience, illustrating the promise of hybrid models that layer interpersonal support onto writing-based reflection [44]. standardised measures such as the PTSD Symptom Scale and FACT were used, often in translated versions, though further validation is warranted to ensure linguistic and cultural fit. Reported challenges included attrition due to time and physical limitations and discomfort with unsupervised writing among participants less inclined to emotional disclosure [43]. Periodic booster sessions, hybrid designs integrating peer support, or prompt structures that emphasise cognitive reappraisal before emotional disclosure may enhance adherence and long-term benefit. Given low infrastructure requirements beyond recruitment, translation, and follow-up, expressive writing is well positioned for dissemination if supported by culturally competent facilitation and optional clinical oversight. Overall, expressive writing may represent a low-cost and scalable psychosocial intervention, particularly for culturally and linguistically diverse survivor populations.

### Nature interventions

Three studies evaluated nature engagement using distinct settings: blue spaces, hospital parks, and urban green environments, and converged on improvements in mental health, quality of life, and functional well-being. A pilot “blue prescription” with sea-side walking, beach bathing, and snorkelling delivered in short sessions reduced tension and anger and increased vigour, with stable physiological markers and effective smartwatch-based monitoring; cost and technical demands may limit scalability and wider public health integration [47]. In a non-randomised controlled programme at a Berlin hospital, both conventional and nature-augmented mind–body day care improved fatigue, quality of life, insomnia, and emotional variables over 12 weeks, but the nature-based arm showed greater gains in quality of life, mindfulness, self-compassion, and lower insomnia at 24 weeks; no adverse events occurred [48]. An integrated urban-park programme in Kyoto that combined forest and horticultural therapy, yoga meditation, and group support improved functional and spiritual well-being, mood, anxiety, and fatigue, and increased natural killer cell activity, suggesting links between psychological resilience and immunological function [49]. The required level of supervision varied: blue space activities demanded water-safety oversight and digital monitoring, while hospital and park programmes were therapist-intensive but did not rely on costly technology. Heterogeneity in measures and designs, with pilot and quasi-experimental approaches predominating, limits generalizability, and cultural validation of instruments remains uneven. Still, the consistent psychological signal across settings supports nature exposure as a viable adjunct to survivorship care.

### Physical activity interventions

Two physical activity studies were included, both of which reflected community-anchored models consistent with SP delivery pathways rather than the broader clinically based exercise oncology literature. A 10-week Nordic Walking programme with weekly 1-h sessions supervised by a certified instructor emphasised technique acquisition, endurance, and strength. Significant gains occurred in the shoulder range of motion for forward flexion, abduction, and external rotation, accompanied by increased vitality, reduced symptom severity, and fewer activity limitations; qualitative interviews underscored valued physical and psychosocial benefits despite a small, uncontrolled sample [51]. A supervised, progressive aerobic programme delivered three times weekly for 4–6 months at community fitness centres achieved improvements in stiffness, fatigue, depression, and multiple quality of life domains, with high adherence of 75–98%, supporting feasibility in real-world settings; small sample size, lack of controls, and a demographically homogeneous cohort temper inference [50]. Together, these studies suggest that structured exercise tailored to treatment sequelae can enhance function and well-being and that community partnerships can facilitate access and adherence within a social prescribing framework.

## Discussion

This scoping review adds to the existing social prescribing (SP) literature by mapping community-anchored and non-clinical interventions that have been implemented for post-treatment cancer survivors and that are operationally consistent with SP pathways. While SP has been widely promoted in primary care and long-term condition management, its application in oncology survivorship remains less clearly synthesised. By focusing on interventions linked through referral or recommendation mechanisms to community resources, this review provides a structured overview of how SP-relevant approaches have been applied in cancer survivorship, the outcomes assessed, and the implementation challenges reported.

Overall, the mapped evidence suggests that SP is a promising instrument for delivering non-medical survivorship support. Across intervention types, benefits were most consistently identified in psychological well-being, coping, and social connectedness. Gardening and nature-based programmes were frequently associated with reductions in distress and fatigue and improved quality of life, while creative, expressive writing, and peer-based interventions supported emotional processing, identity reconstruction, and reduced isolation. Physical activity and yoga-based interventions were associated with improvements in function, sleep, and mood. Taken together, these findings indicate that SP-relevant interventions can address multiple survivorship domains, including psychological, physical, and social well-being, aligning with the holistic needs of cancer survivors.

However, the evidence base remains constrained by significant heterogeneity in intervention design, outcome measurement, and reporting of SP delivery pathways. Many studies did not provide detailed descriptions of intermediary SP components such as structured needs assessment, formal referral pathways, link-worker involvement, or implementation fidelity. As a result, while the included interventions were community-based and linked through referral or recommendation mechanisms, the extent to which they reflect fully developed SP models varies across settings. This highlights a key gap in SP research in oncology survivorship: interventions may deliver SP-like benefits but are often not described or evaluated using consistent SP implementation frameworks.

Several cross-cutting facilitators were identified. Community-embedded interventions appeared to promote acceptability by providing survivors with accessible, non-clinical environments that support autonomy and normalisation beyond the healthcare system. Group-based formats (peer support, yoga, arts programmes) supported social connection and engagement, while home-based interventions such as gardening reduced travel burden and may be particularly beneficial for survivors with fatigue, mobility limitations, or rural residence. Interventions that incorporated structured facilitation (e.g. mentoring, trained instructors, peer leaders) also tended to report stronger engagement and more clearly defined outcomes.

Conversely, several barriers may limit the implementation and scalability of SP for cancer survivorship. Resource intensity constrains scalability for programmes needing specialised staff, equipment, or facilities, such as blue-space protocols with smartwatch monitoring [47], oncology-competent yoga or Nordic walking instructors [51], and licenced art therapists [39]. Gardening and broader nature activities are adaptable and potentially lower-cost but depend on access to safe green or coastal spaces and sustained participation. Even highly adherent gym-based programmes rely on durable community partnerships that may be difficult to maintain over time [50]. Structured interventions yield more consistent and quantifiable outcomes but impose scheduling rigidity that can be difficult for survivors with fluctuating energy, fatigue, or treatment-related complications. Study design limitations also constrain certainty: many investigations used feasibility or pilot designs, small samples (for example, 22 to 28 participants in nature and Nordic Walking studies) [49–51], non-randomised or cross-sectional methods [29, 30, 37], limited or no active control conditions [36], and short follow-up, making durability of effects unclear. Technology-enabled protocols raise issues of data reliability, burden, and cost. Most notable is the predominance of breast cancer cohorts, reflecting incidence patterns and advocacy landscapes but limiting applicability to survivors with different prognoses, symptoms, and psychosocial needs [1, 53, 54]. Cultural considerations were unevenly addressed: beyond Spanish-language yoga for Latinas [32] and Indigenous art therapy [40], few studies probed how culture, language, spirituality, or social norms around emotional expression shape engagement and outcomes. Participant heterogeneity in baseline physical capacity, comfort with expressive types, and willingness to disclose emotions complicates standardisation, while attrition often skews toward higher-functioning individuals [28, 51].

One underexplored avenue is volunteering as an intervention. Transitioning survivors from recipients to contributors, through peer mentoring, garden leadership, or group facilitation, could restore agency, deepen purpose, and sustain engagement. Integrating structured volunteer roles into social prescribing may strengthen reciprocity and programme durability while building community capacity. Future work should test how volunteering intersects with recovery trajectories and resilience and evaluate cost-effectiveness, reach, and equity impacts within survivorship ecosystems.

Although this review emphasises evidence synthesis, training and implementation considerations remain important for translation into practice. Personnel should follow standardised protocols that specify objectives, content, and techniques, supported by competency-based training that combines theory and supervised practice tailored to survivorship needs. Cultural competence, ethical practice, and trauma-informed skills are essential for managing sensitive topics, facilitating disclosure safely, and navigating referral pathways. For activity-based interventions, instructors require training in cancer-specific screening, adaptation, and risk management, including emergency procedures for outdoor programmes. In settings with limited resources, simplified assessment tools and digital data collection can maintain evaluation quality and scalability. These training elements are foundational to safety, consistency, and fidelity and are summarised in Table 2.

**Table 2:** Key implementation and safety considerations for social prescribing intervention types

Intervention type	Key implementation requirements	Key safety considerations
Gardening	Access to gardening space and tools; basic horticultural guidance	Injury prevention; tool safety; environmental hazards
Peer support	Trained peer facilitators; meeting space or online platform	Confidentiality; emotional boundaries; referral for distress
Yoga/spiritual	Suitable space; trained instructor; basic equipment	Contraindications; modification for symptoms/limitations
Creative arts	Materials and safe facilitation environment	Emotional distress; safeguarding and referral pathways
Expressive writing	Written or digital materials; clear prompts	Emotional distress; signposting support services
Nature-based	Safe outdoor setting; supervision where needed	Risk management; weather exposure; emergency procedures
Physical activity	Qualified exercise professional; accessible venue	Screening; injury prevention; adverse event management

A key strength of this scoping review is its broad mapping approach, which captures a wide range of community-based and non-clinical interventions relevant to social prescribing (SP) in cancer survivorship. However, this breadth also represents an important limitation, as heterogeneous reporting and inconsistent description of key SP elements limit the ability to draw strong conclusions about SP as a distinct and standardised model of care. In particular, many included studies did not comprehensively report intermediary SP components such as structured assessment processes, formal referral pathways, or the involvement of link workers. As a result, although the interventions included in this review were community-anchored and linked through a referral or recommendation mechanism, the extent to which they reflect fully developed SP models may vary across settings, highlighting an important gap in reporting standards for SP interventions in oncology survivorship research. In addition, despite the extensive literature on exercise, peer support, and expressive writing in cancer survivorship, only a small number of studies in these categories met inclusion criteria because we required interventions to be community-anchored and linked through a structured referral or recommendation mechanism consistent with SP, thereby excluding many clinic-based, rehabilitation-centred, or purely trial-based programmes and potentially omitting interventions such as the Australian *My Changed Body* study where a referral-based SP pathway was not clearly reported [55]. Small samples reduce power and heighten selection bias, as seen when peer support participants outperform population norms at baseline [31]. Non-randomised designs and a dearth of long-term follow-up impede causal inference and knowledge of durability, with exceptions in gardening and expressive writing where more rigorous designs have begun to appear [22, 23, 42–46]. Publication bias may inflate positive signals, and inconsistent outcome measures hinder cross-study comparison. Future trials should prioritise larger, diverse cohorts; standardised, validated measures with cultural and linguistic adaptation; active comparators; and follow-up of at least 6 months to assess maintenance. Hybrid and digital models could extend reach, including tele-mentored gardening, virtual or asynchronous expressive writing with optional facilitated sessions, app-supported art-making, and wearable-informed home-based activity. Urban gardening and virtual nature exposure could mitigate environmental barriers. Integrating professional input into peer programmes, while preserving peer identity, may improve safety and retention. Cost-effectiveness analyses are overdue to inform payer and policy uptake.

Policy alignment can amplify impact by embedding SP within survivorship pathways, promoting civic participation, and expanding access to culturally meaningful, non-medical supports. Collaboration between healthcare systems, policymakers, and the voluntary sector will be essential to ensure sustainable financing, workforce development, and equitable provision across regions and populations.

## Conclusions

This scoping review highlights the range of community-based and non-clinical interventions that have been described within, or are operationally consistent with, social prescribing (SP) for post-treatment cancer survivors. The mapped evidence suggests that interventions such as gardening, peer support, expressive writing, creative arts, yoga/spiritual care, nature-based activities, and community physical activity programmes may improve psychological well-being, social connectedness, and quality of life outcomes. However, important uncertainties remain regarding SP as a distinct and standardised model of care, as many studies provided limited reporting of key SP pathway elements such as structured assessment, formal referral mechanisms, and link-worker involvement. In addition, evidence on long-term outcomes, implementation effectiveness, sustainability, and cost-effectiveness remains limited. Future research should prioritise clearer reporting of SP delivery pathways, evaluation of implementation models (including link-worker roles), and robust trials with longer follow-up to determine the effectiveness and scalability of SP within cancer survivorship care.

## Supplementary Information

Below is the link to the electronic supplementary material.

ESM 1 Supplementary Material 1 (DOCX 13.8 KB)

## Data Availability

No datasets were generated or analysed during the current study.
