# Supplementary material for: Social prescribing for people recovering from treatment for cancer: a systematic scoping review
Source: Support Care Cancer. 2026 Apr 25;34(5):464. doi: 10.1007/s00520-026-10561-w (PMC13110229; doi:10.1007/s00520-026-10561-w)
Supplement: Supplementary file 1 — Supplementary Material 1 (DOCX 13.8 KB) [file 520_2026_10561_MOESM1_ESM.docx]

**Supplementary 1**

**Search Terms**

("well-being"[Title/Abstract] OR "Quality of Life"[Title/Abstract] OR "Psychological Well-Being"[MeSH Terms] OR "Quality of Life"[MeSH Terms]) **AND** ("social prescribing"[Title/Abstract] OR "social prescription"[Title/Abstract] OR "social referral"[Title/Abstract] OR "community referral"[Title/Abstract] OR "community prescription"[Title/Abstract] OR "community connection"[Title/Abstract] OR "linking scheme"[Title/Abstract] OR "art prescription"[Title/Abstract] OR "nature prescription"[Title/Abstract] OR "garden*"[Title/Abstract] OR "museums"[Title/Abstract] OR "public facilities"[Title/Abstract] OR "recreational facilities"[Title/Abstract] OR "cultur*"[Title/Abstract] OR "park"[Title/Abstract] OR "volunteering"[Title/Abstract] OR "blue prescri*"[Title/Abstract] OR "green prescri*"[Title/Abstract] OR "Social Support"[MeSH Terms]) **AND** ("cancer"[Title/Abstract] OR "oncolog*"[Title/Abstract] OR "neoplas*"[Title/Abstract] OR "tumo*r"[Title/Abstract] OR "malign*"[Title/Abstract] OR "carcinoma"[Title/Abstract] OR "Neoplasms"[MeSH Terms] OR "Cancer Survivors"[MeSH Terms]).
